# Image Analysis Semi-Automatic System for Colony-Forming-Unit Counting

**DOI:** 10.3390/bioengineering9070271

**Published:** 2022-06-22

**Authors:** Pedro Miguel Rodrigues, Jorge Luís, Freni Kekhasharú Tavaria

**Affiliations:** CBQF—Centro de Biotecnologia e Química Fina—Laboratório Associado, Escola Superior de Biotecnologia, Universidade Católica Portuguesa, Rua de Diogo Botelho 1327, 4169-005 Porto, Portugal; s-jmbluis@ucp.pt

**Keywords:** colony forming units, petri-plates, image processing, enumeration

## Abstract

Background: Accurate quantitative analysis of microorganisms is recognized as an essential tool for gauging safety and quality in microbiology settings in a wide range of fields. The enumeration process of viable microorganisms via traditional culturing techniques are methodically convenient and cost-effective, conferring high applicability worldwide. However, manual counting can be time-consuming, laborious and imprecise. Furthermore, particular cases require an urgent and accurate response for effective processing. Methods: To reduce time limitations and discrepancies, this work introduces an image processing method capable of semi-automatically quantifying the number of colony forming units (CFUs). This rapid enumeration technique enables the technician to provide an expeditious assessment of the microbial load of a given sample. To test and validate the system, three bacterial species were cultured, and a labeled database was created, with subsequent image acquisition. Results: The system demonstrated acceptable classification measures; the mean values of *Accuracy*, *Recall* and *F-measure* were: (1) 95%, 95% and 0.95 for *E. coli*; (2) 91%, 91% and 0.90 for *P. aeruginosa*; and (3) 84%, 86% and 0.85 for *S. aureus*. Conclusions: Evidence related to the time-saving potential of the system was achieved; the time spent on quantification tasks of plates with a high number of colonies might be reduced to a half and occasionally to a third.

## 1. Introduction

The assessment of bacterial growth (enumeration) is one of the main, basic and frequent requisites when addressing studies on microorganisms. Evaluation of food and drug safety and the control of environmental quality are examples of fields that rely on the measurement of microorganism survival and proliferation rates. Due to its importance, this evaluation has been and will continue to be widely employed worldwide on a daily basis. Therefore, the improvement of the available techniques and practices is appreciated. The abovementioned processes often require the counting of bacteria in a unit volume of bacterial broth, and there are several methods available that can achieve it in a direct or indirect way: membrane filtration, turbidity, metabolic activity, dry weight and the viable cell counting as indirect measures, and microscopic counts and cytometry, as direct measures.

Of these methods, the viable cell counting method is the most cost-effective, easily-accessed method to evaluate culturable microbial loads. When performing this method, the viable number of bacteria can be obtained by counting the number of colonies on the plate. However, the manual counting of colonies is laborious, time-consuming and error-prone. In addition, it is not immediate, requiring the incubation period for colony growth. Automated bacterial counters are also available as fully automated counting systems but can be highly expensive and not affordable for small labs, or multiple instruments may be required for large facilities and research centers [1,2].

Image processing techniques are thus seen as a potential option for performing the counting tasks. Several reported studies do exist in the literature reporting image analysis in microbiology. Some use computer image analysis (CDIA) as one of the operations for a more general procedure called computer digital image processing (CDIP) [3]. According to the state of the- art related to this subject, several image processing software programs have also been developed. In [1], a low-cost, high-throughput colony counting system consisting of a colony counting and a consumer-grade digital camera was created and proposed. The software, called NICE, can count bacterial colonies as part of a high-throughput multiplexed opsonophagocytic killing assay used to characterize pneumococcal vaccine efficacy. It reads standard image formats and therefore may be used in conjunction with other imaging systems. Clono-Counter, developed in [4], is a colony counting software through the use of three parameters, i.e., maximum size of the colony, gray levels and gray level distribution. Despite provided guidelines, the users need to have some experience to find the correct parameters. A fully-automated colony counter for bacterial colony enumeration was proposed in [5] with considerable accuracy to detect colonies cultivated in colored media but has limitations with those with transparent media. Two years later, the same authors developed a method to allow enumerations in a wider range of media with a reasonable performance on both colored and translucent media [5]. In [6], a CCD (Charged-coupled Device) camera was used to capture images of colonies; images were treated as perfect circles, removing the boundary and consequently the linked colonies, underestimating the total number of bacteria. However, the results were highly correlated with the ones gathered from manual counts. Martinez-Espinosa et al. [7] also used a commercial CCD to record the images of colonies cultivated in petri dishes and using an open source software to process them concluded that better reliability of the results were attained when compared to visual counting. OpenCFU was also developed, where the control over the processing parameters is provided and thus could be used in applications in colony counting and other circular objects, yet, the process lacks a higher level of automation [1,8,9]. Image processing has further been used as an alternative method in solving other specific biological challenges such as counting mammalian cell colonies [10,11] or monitoring the growth of cancer cells [12]. The application and correct integration of mathematical algorithms, boundary selection, filters distance transforms, segmentation, pattern recognition and object labelling can modify a digital image to extract valuable information from a region of interest for a very specific purpose [5,7,13,14].

Some of the outlined existent solutions exhibit excellent accuracy results and are seen as potential solutions that contribute to the laboratory automation and efficiency. However, most of them reveal one of two flaws: excessive user-provided action or information, and statistical relevance, expressing the need of being tested in larger and significant databases, resembling the actual and demanding laboratory environment. Furthermore, caution should be taken when interpreting the results of colony-forming units (CFU) in Petri plates, making sure the microbial species under study grow as individual cells and do not form aggregates in natural conditions. However, even in such cases, partial aggregation may be induced by laboratory practices and manipulations [15]. Moreover, the external appearance of the colonies on solid media is one of the main phenotypic characteristics in microbial identification [16]. Therefore, differences in colony appearance serve to differentiate microorganisms at the level of various taxonomic groups, including at the single species level and are reliant on cultivation conditions, medium composition, temperature and growth time [17]. Although this cannot be the sole parameter used to identify microorganisms, it may be used with reliance in screening for purity of the cultures, disease diagnostics, sanitary inspection and epidemiological expertise areas [3]. Table 1 summarizes image analysis-based methods for bacterial enumeration published to date.

With this work, we intended to complement the knowledge by developing a fast software capable of semi-automatically quantifying the number of colonies in Petri plates with very low user-provided action and with high levels of classification indexes for a selected set of bacterial species.

In terms of structure, this paper is organized in four main sections. In Section 2, the methodology concerning bacterial cultures, image acquisition, image processing and the enumeration process is explained. The obtained results and the inherent discussion were covered in Section 3. Lastly, some conclusions are drawn in Section 4.

## 2. Methodology

In this section, all developed procedures are described. Primarily, the microbiology methods performed to acquire the image database are detailed, and subsequently the flowchart of the enumeration process is presented step-step.

### 2.1. Bacterial Cultures

Three bacterial species, *Escherichia coli*, *Pseudomonas aeruginosa* and *Staphylococcus aureus*, were used for the image database attainment. These were provided from the internal collection of CINATE (Center for Innovation and Technological Support, ESB, UCP) and were cultivated aerobically at 37 °C.

#### 2.1.1. Culture Media

Two general-purpose media were used to grow the 3 bacterial species, Brain-Hearth Infusion Broth^™^ and Trypto-Casein Soy Agar^™^, both from BIOKAR Diagnostics©, Allonne, France.

#### 2.1.2. Inoculum Preparation

Frozen cultures of the 3 bacterial species cryopreserved in mini-cryovials at −80 °C, were thawed via gentle agitation in a water bath that was set to the normal growth temperature of each bacterial species, with prior decontamination of the outer surface using 70% ethanol. The entire content of each vial was transferred to sterilized test tubes containing the growth media. The 3 cultures were incubated overnight at 37 °C. Cultures were then streaked onto agar plates to produce isolated/purified colonies. With evidence of isolated and pure colonies and using the inoculation loop, a single colony was collected and transferred to a sterilized test tube containing 10 mL of fresh media. The new pre-inoculum was incubated overnight under 37 °C. Subsequently, 100 μL were transferred from the preceding solution to a 10 mL test tube containing fresh media and allowed to grow overnight at 37 °C—the final inoculum was achieved.

#### 2.1.3. Serial Dilutions

After mixing, 1 mL of the inoculum was added to a sterilized eppendorf and a centrifugation step was performed. The solutions were centrifuged twice for 10 min at 5000× *g*, each time resuspended in 1 mL of a Ringer solution previously prepared. The content of the 3 microorganism solutions was then transferred to sterilized test tubes containing 9 mL of Ringer, and serial decimal dilutions were made.

#### 2.1.4. Spread Plate Method

**Figure 1:**
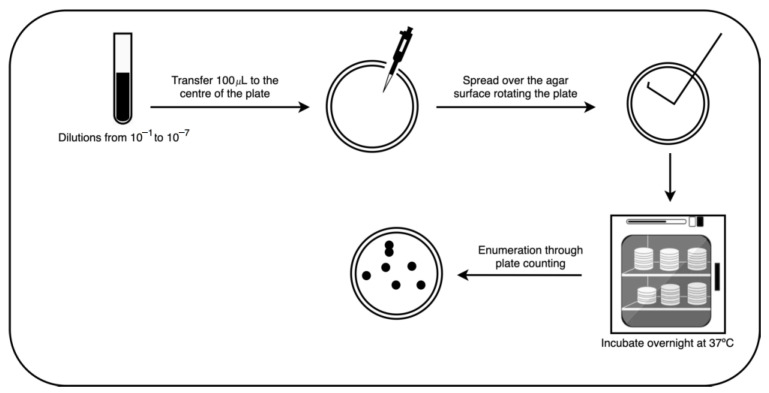
Spread plate method.

For enumeration; the spread plate method was employed; 100 μL of each dilution were pipetted onto the center of the surface of the sterilized agar plate and spread. All samples used were plated in triplicate; the plates were then incubated overnight at 37 °C (see Figure 1 for more details).

Colony enumeration was performed through the plate count method. The number of colonies was recorded and posteriorly attributed to each image of the database.

### 2.2. Image Database Collection

**Figure 2:**
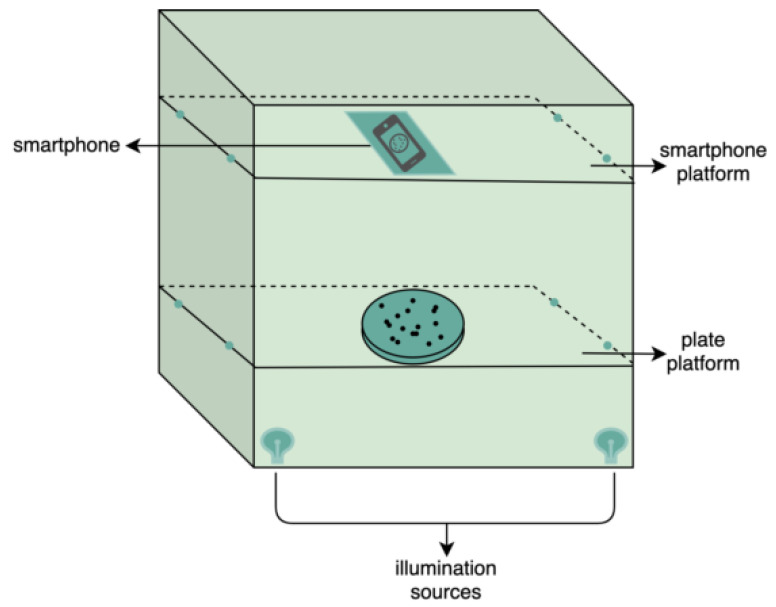
Scheme of the handled black box to image acquisition.

For image database collection, each manual counting was executed and registered, and the plates were ready for image capture. The image acquisition system used in this study, i.e., a black card box, is shown in Figure 2. To accentuate the region of interest and provide adequate contrast between the colonies and the background, the 9 cm plate was illuminated from below by two sources of yellow light, aiming for uniformly distributed illumination. A transparent LDPE platform was used to fix the position of the plate. The main purpose of the design of this apparatus was to obtain the images inside the periphery of the plate, including the edge of the periphery. The external light was blocked with the cover of the box, and a PDAF smartphone camera with 12 megapixels (3024 × 4032) was used to capture the images. Another platform was included to always enable the placement of the smartphone in the same location. With the designed and simple apparatus, good quality images were obtained. Finally, the database was cataloged, attributing number, bacterial species and total manual count to each image, e.g., *CEMTimages_0278_EC_C76*, where *0278*, *EC* and *C76* meaning the image number, bacterial species and total count, respectively. The built dataset consisted of about 1150 labeled images with approximately the same number of images for each bacterial species to attain a balanced database.

After an analysis of the database, a set of characteristics were outlined to select the techniques to be implemented; a high amount of noise, reduced contrast between fore and background, existing clumps of bacteria, variation of colony size, presence of high intensity areas and inherent plate marks were some of the aspects taken into account. Thus, there was a special concern to develop a set of procedures capable of overcoming the emerged and enunciated adversities, achieving the best results possible. Moreover, there was a concern in building a versatile algorithm, i.e., applicable to images with different properties or acquired through distinct laboratory procedures.

### 2.3. Semi-Automatic Enumeration Process Method

**Figure 3:**
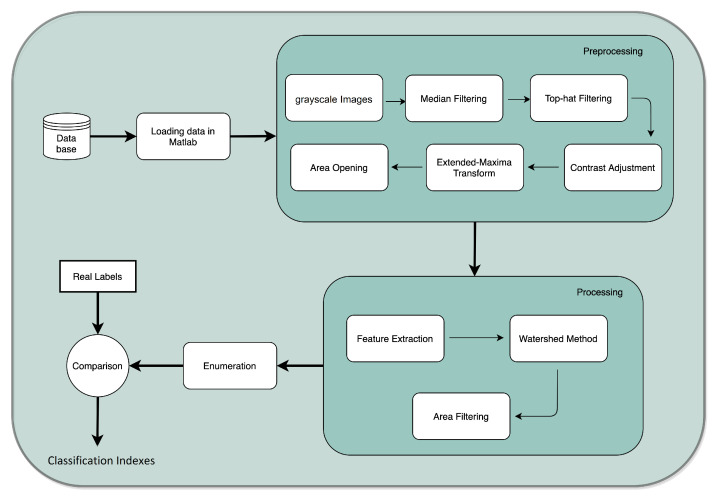
Flowchart of the semi-automatic enumeration process.

The database was first loaded in MATLAB^®^ software (Mathworks, Inc., Natick, MA 01760-2098, USA). The developed algorithm performs three major steps: (1) preprocessing; (2) processing; and (3) enumeration. Before the preprocessing begins, the user is asked to input a parameter, i.e., the minimum size of a colony. This action is performed by an interactive placement of an ellipse around the minimum-sized colony of the plate. It is worth acknowledging that during the overall process, this is the only occasion when the user is asked to input information. Therefore, the computed result is compared with the real labels from each image, and consequently a set of classification indexes are computed. The process is outlined in Figure 3.

#### Preprocessing

**Figure 4:**
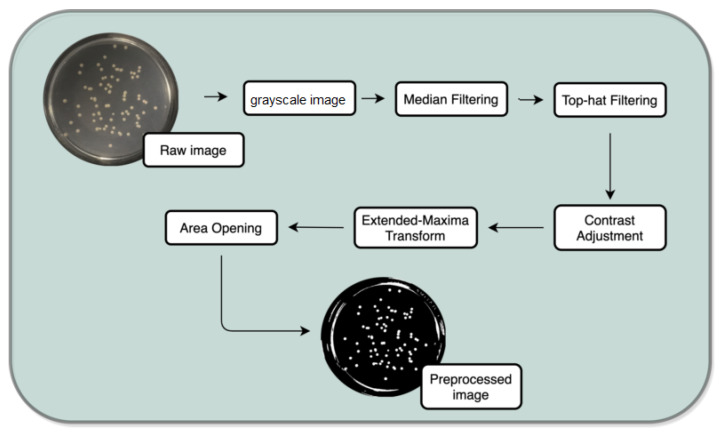
Preprocessing task.

This task aims to produce the most satisfactory and noise-free images possible to further the segmentation in the following stage (see Figure 4).

**Figure 5:**
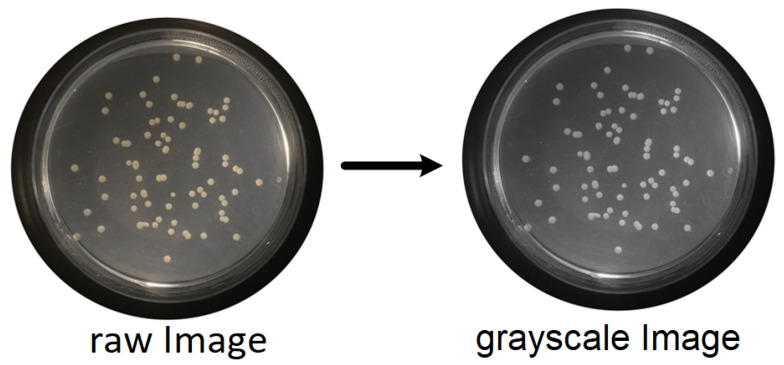
Conversion of raw color image to grayscale image.

**Figure 6:**
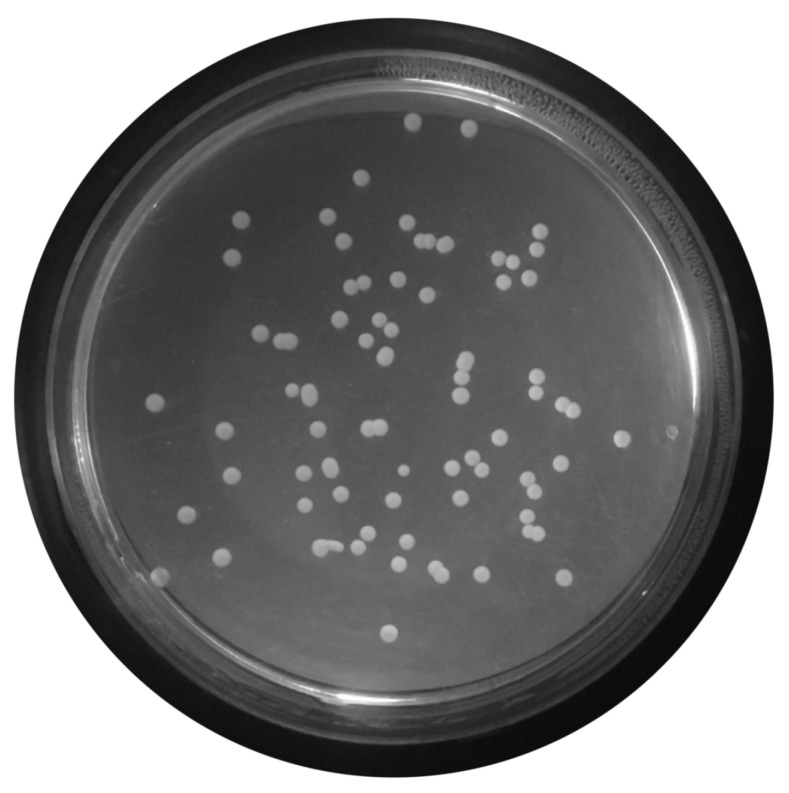
Median-filtered image.

**Figure 7:**
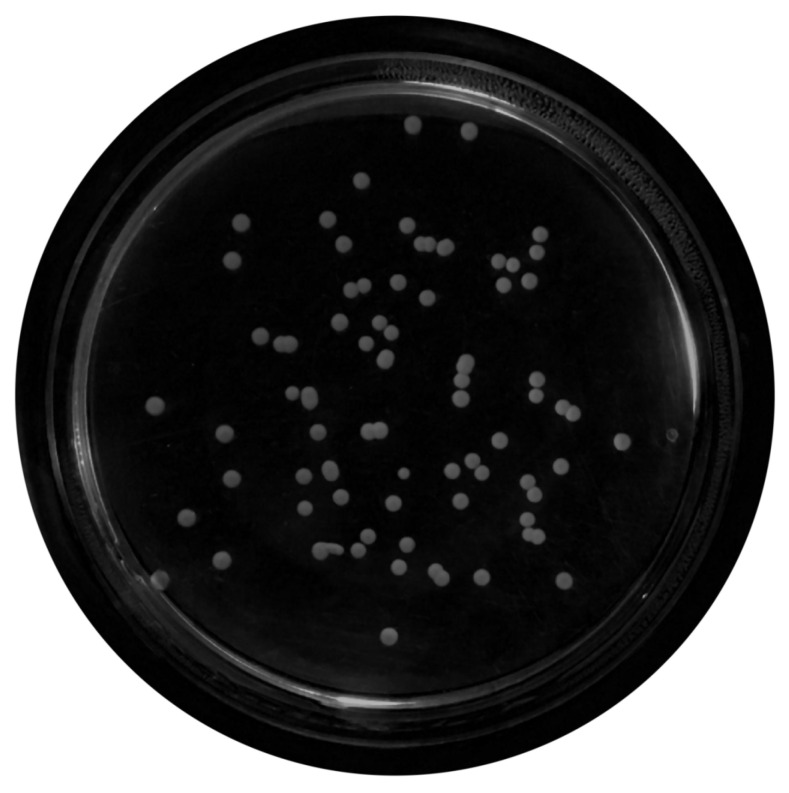
Top-hat transform with a disk structuring element.

**Figure 8:**
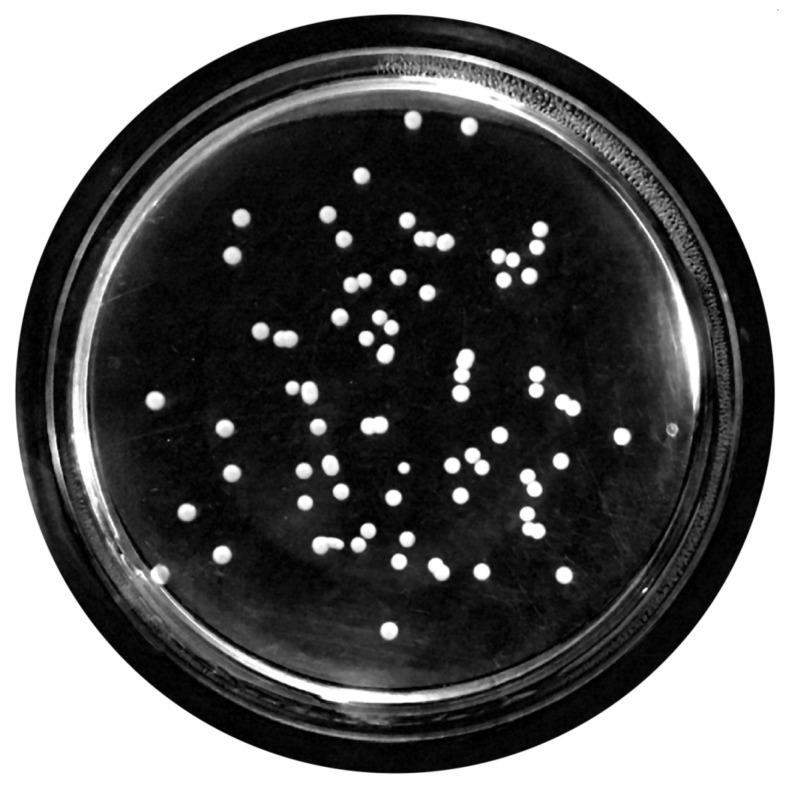
Contrast adjustment.

**Figure 9:**
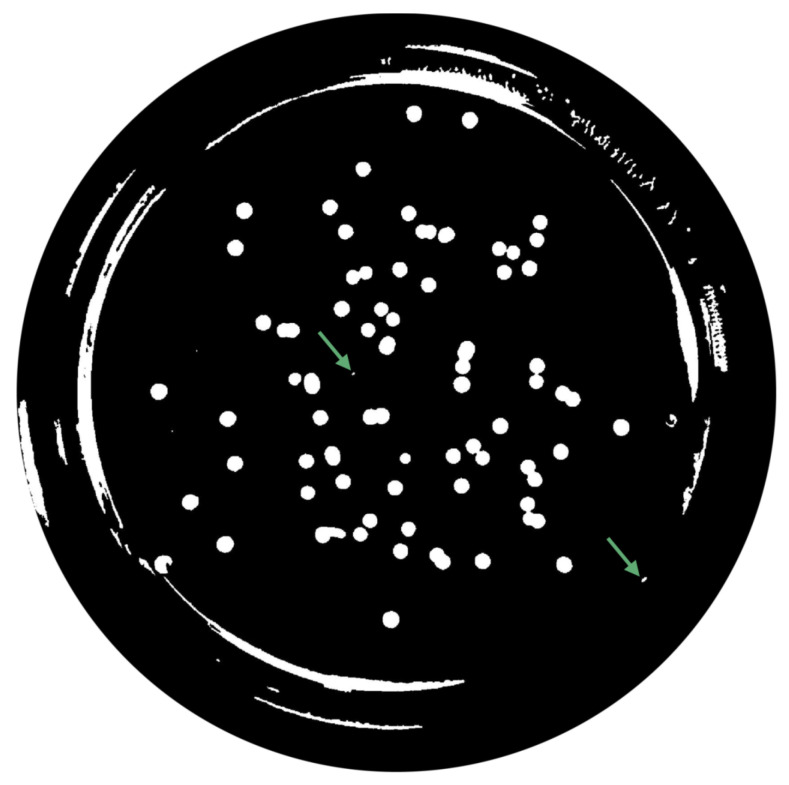
Extended-Maximum Transform.

**Figure 10:**
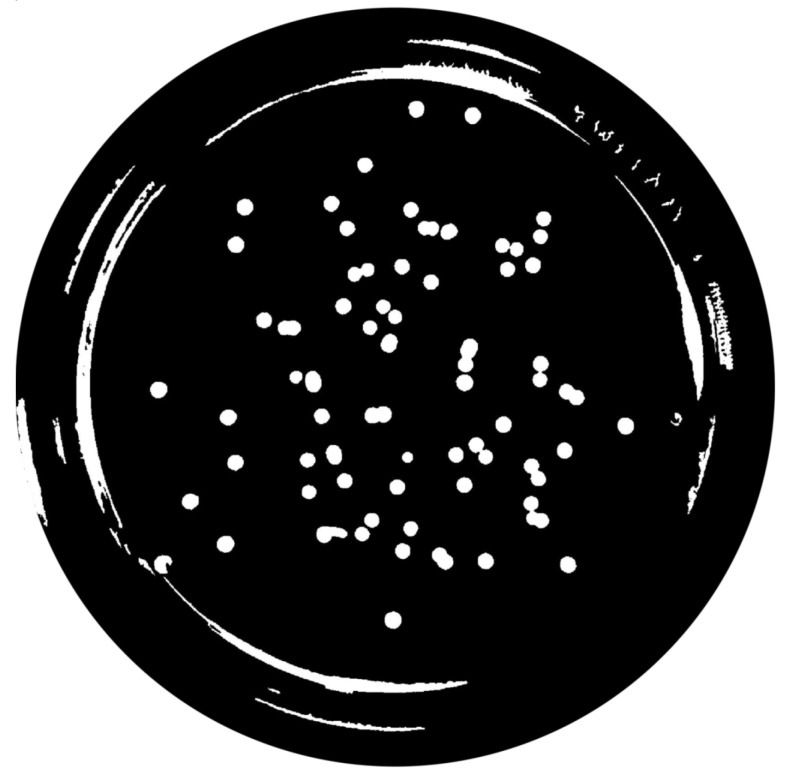
Elimination of small objects with less than 250 pixels by area-opening operation.

For this propose, a set of filters and transformations were applied to the original images in the following sequence:

- RGB (red, green, blue components-color) images to grayscale images:
  All database raw RGB color images have been converted to grayscale images by using the National Television System Committee (NTSC) formula [28]: 0.299 * red component + 0.587 * green component + 0.114 * blue component, view Figure 5.
- Median Filtering:
  This technique is widely applied in image processing algorithms for reducing the noise without a contrast loss [29]; a 9 × 9 kernel median filter was adopted (see the result in Figure 6).
- Top-Hat Filtering:
  To correct possible uneven illumination, which leads to uneven contrast, a top-hat transform with a 200-pixel radius and disk-shaped single structuring element was performed. This morphological filter computes the image opening and then subtracts the image result from the input image [30], in this case the median-filtered image. Figure 7 illustrates the result of this step.
- Contrast Adjustment:
  In this step (Figure 8), the image was normalized according to Equation (1); $p_{\mathrm{adjusted}}$ is denoted as the replaced pixel value, *p* the current pixel value, $p_{\min}$ the minimum pixel value, NC the normalization coefficient and MI the maximum intensity value of the image [31].
  (1) $$\sum_{p_{n}}p_{\mathrm{adjusted}}=\frac{p-p_{\min}}{NC}\times MI$$
- Extended-Maxima Transform:
  Another normalization process occurred; the extended-maxima transform was applied, where the intensities of points inside the foreground regions were changed to show the distance to the closest boundary from each point. First the regional maxima were found; objectively, 80 pixel-region maxima were computed and with 8-connected pixels, i.e., the neighborhood of a pixel is the adjacent pixels in the horizontal, vertical or diagonal direction; subsequently, the transformation is performed (see Figure 9).
- Area Opening:
  The intent of this stage was the removal of small objects from the image (objects marked in Figure 9 with green arrows). All connected components with fewer than 250 pixels were removed. The result is presented in Figure 10.

### 2.4. Processing

**Figure 11:**
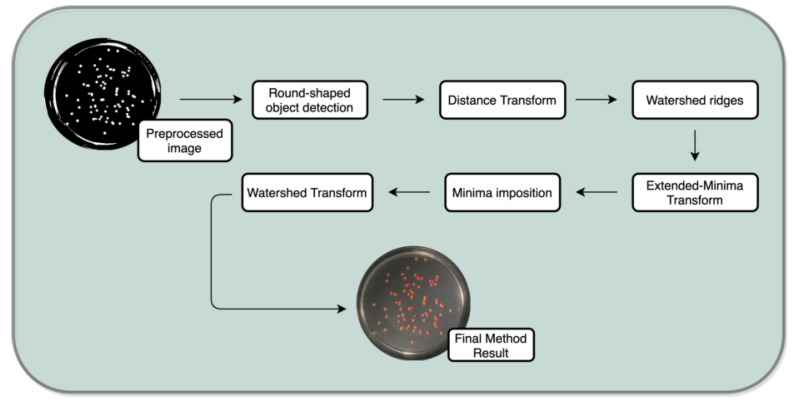
Processing task.

The second main step of the process–processing is subdivided in two stages; first the detection of round-shaped objects and after that the segmentation of the detected round-shaped objects is performed. The objective at this stage is to establish isolated colonies to perform an efficient enumeration process (view Figure 11 for more details).

**Figure 12:**
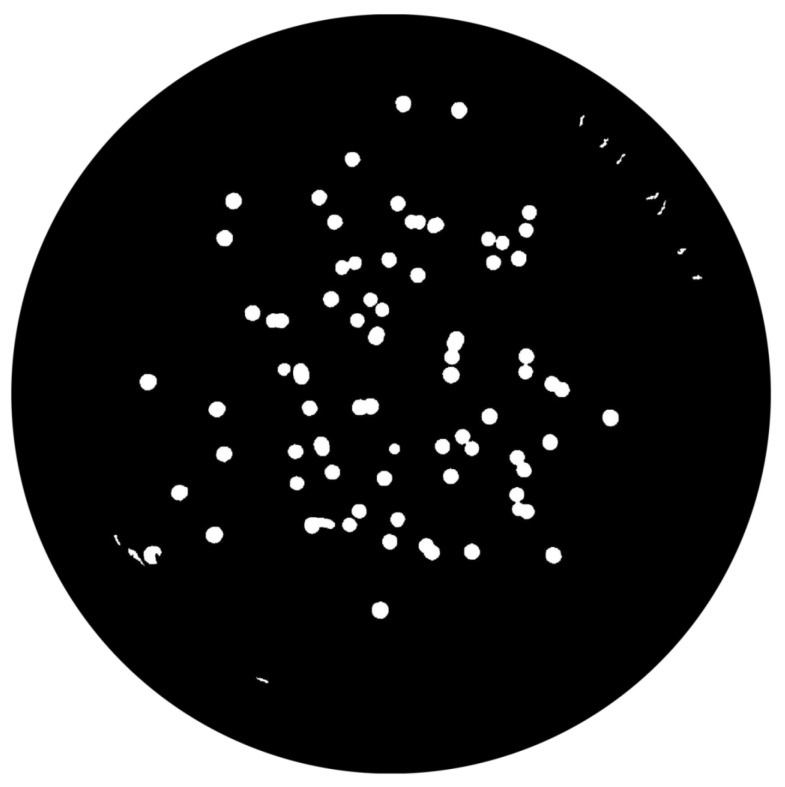
Detected round objects.

**Figure 13:**
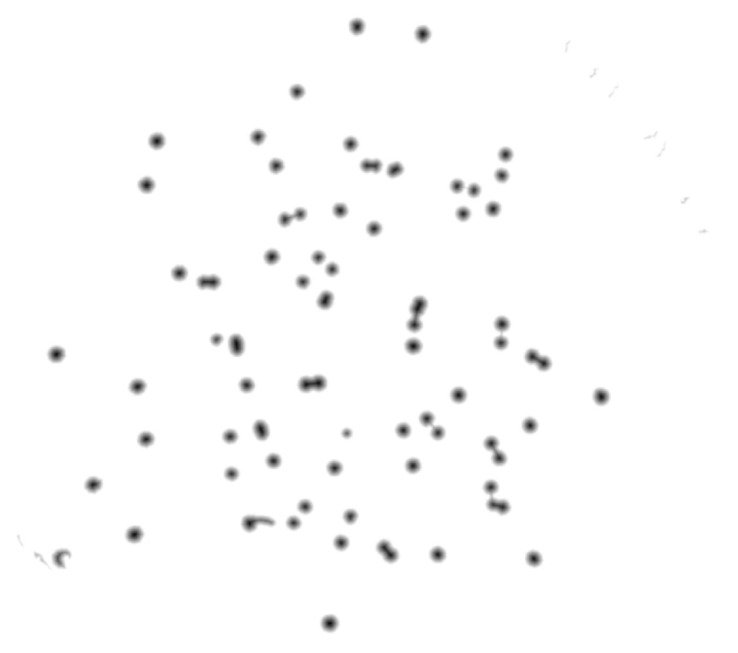
Negative of distance transform image result.

**Figure 14:**
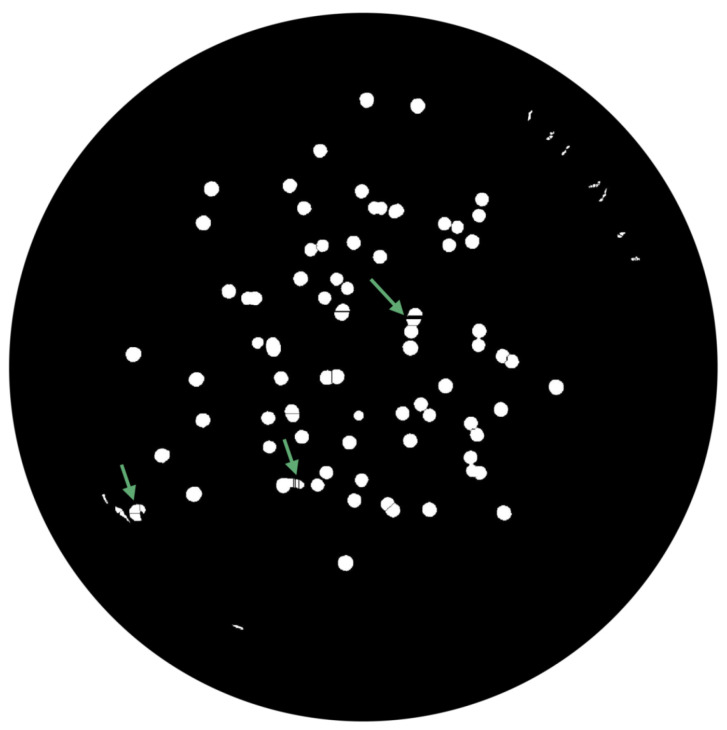
Segmentation through watershed ridges.

**Figure 15:**
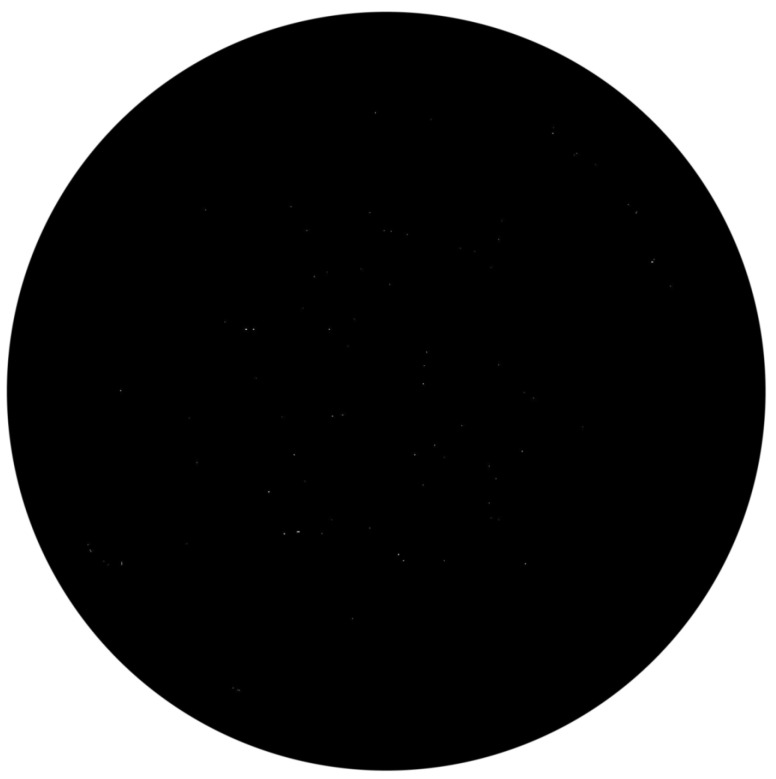
Extended-Minimum Transform, assigning small dots to each “true” minima.

**Figure 16:**
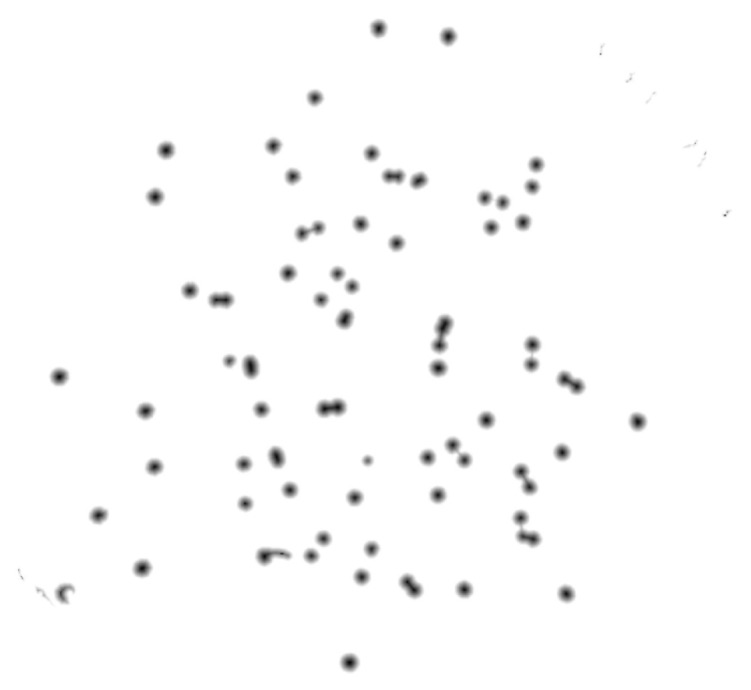
Minima imposition.

**Figure 17:**
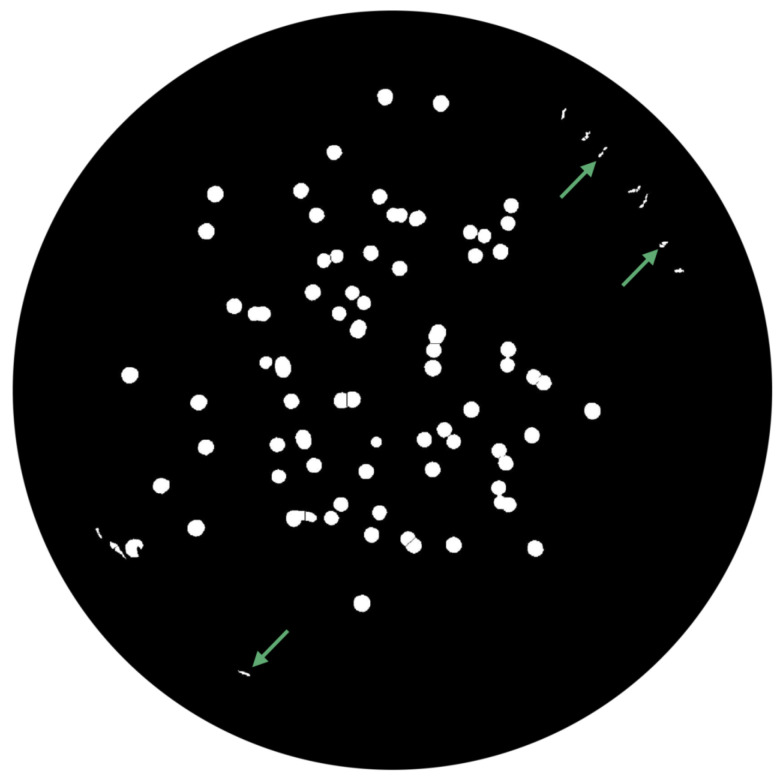
Segmented colonies by watershed transform.

**Figure 18:**
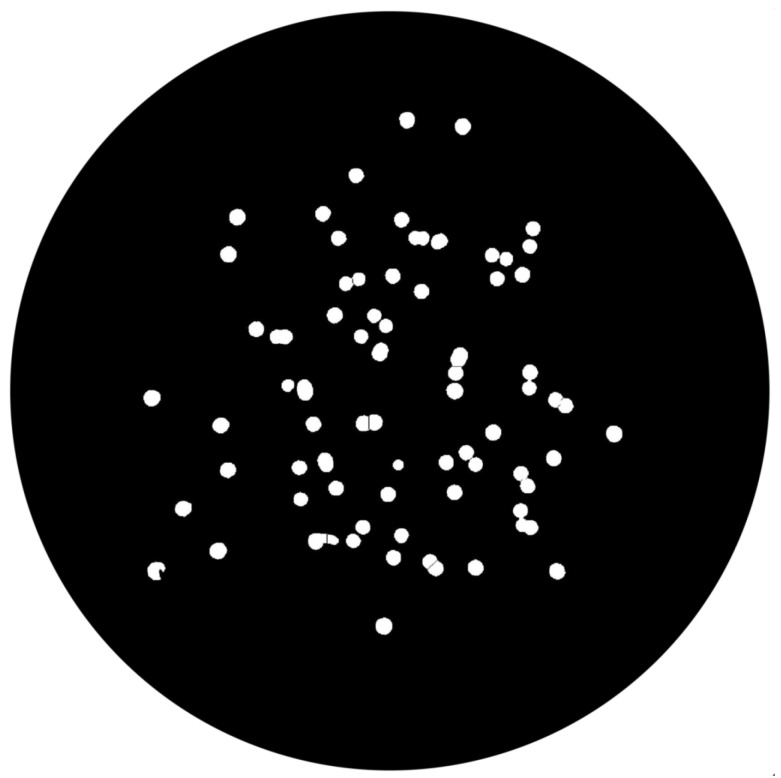
Area filtered image.

- Round-shaped objects detection:
  The aim of this task is to select potential colonies. Foremost, from all the detected objects, 3 features are extracted: *area*, *perimeter* and *circularity*. From the areas and perimeters, a metric of the “roundness” of the objects is computed, and those objects with a value of 1 are indicative of perfect circles. Since several colonies are not a perfect circle, not only the objects with a metric of 1 are selected but also those within an interval, i.e., 0.48 to 1.6. The third extracted feature, circularity, is also a metric of “roundness” and improves the detection procedure (check task image result at Figure 12).
- Watershed Method:
  This phase is important as it transforms the previous image into one where the objects are catchment basins—watersheds, to posteriorly being segmented. The watershed transform is only performed at this stage to avoid over-segmentation issues. This process is subdivided in 5 sub-steps: distance transform, watershed ridges, extended-minima transform, minima imposition and finally the watershed transform itself [32].
  - Distance Transform
    At this step, the distance transform is computed, i.e., the distance from every pixel to the nearest non-zero-valued pixel. However, to turn bright areas into catchment basins and to assign one catchment basin to each object, the distance transform has to be negated (check the example result in Figure 13).
  - Watershed ridges
    Upon performing, the following operation is intended to segment the colonies using the watershed ridges, and these values correspond, in fact, to zero; thus, if zero is assigned to those values, they become background pixels and subsequently split the colonies. The effects that the aforementioned operation produces are displayed in Figure 14 and identified by green arrows.
  - Extended-Minima Transform
    As can be seen in Figure 14, the watershed function tends to perform over-segmentations (every local minimum becomes a catchment basin); thus, it is necessary to filter meaningless local minima. Small dots are assigned to each colony, and the outcome is shown in Figure 15 [33].
  - Minima imposition
    The marker image $f_{m}$ can be defined for each pixel *p*, as follows,
    (2) $$f_{m}\left(p\right)=\left\{\begin{array}{cc} 0, & \mathrm{if}p\mathrm{belongs}\mathrm{to}a\mathrm{marker}\;;\\ t_{max,} & \mathrm{otherwise}\;. \end{array}\right.$$
    The minima imposition of the input image is then performed in two steps: (1) the pointwise minimum between the input image and the marker image is computed: $f\wedge f_{m}$. Through the latest, minima are created at locations corresponding to the markers. Moreover, two distinct minima to impose may fall within a minimum of *f* at level 0; therefore it is necessary to consider $\left(f+1\right)\wedge f_{m}$ rather than $f\wedge f_{m}$; (2) morphological reconstruction by erosion of ($\left(f+1\right)\wedge f_{m}$) from the marker image $f_{m}$:
    (3) $$R_{\left(f+1\right)\wedge f_{m}}^{\varepsilon }\left(f_{m}\right)\;;$$
    the extended transform is then changed in a way that no minima occur in the previously filtered locations. The output of this step is displayed in Figure 16 [33].
  - Watershed Transform
    Finally, the watershed transform is performed using the watershed function, and an image with the segmented colonies is exhibited (Figure 17).
  Area Filtering
    At this stage, it is necessary to remove artifacts and outliers (pointed in Figure 17 by green arrows) on images. All the connected components outside of a specified range are extracted, returning an image containing only those objects that meet the criteria, depicted in Figure 18.

### 2.5. Enumeration

**Figure 19:**
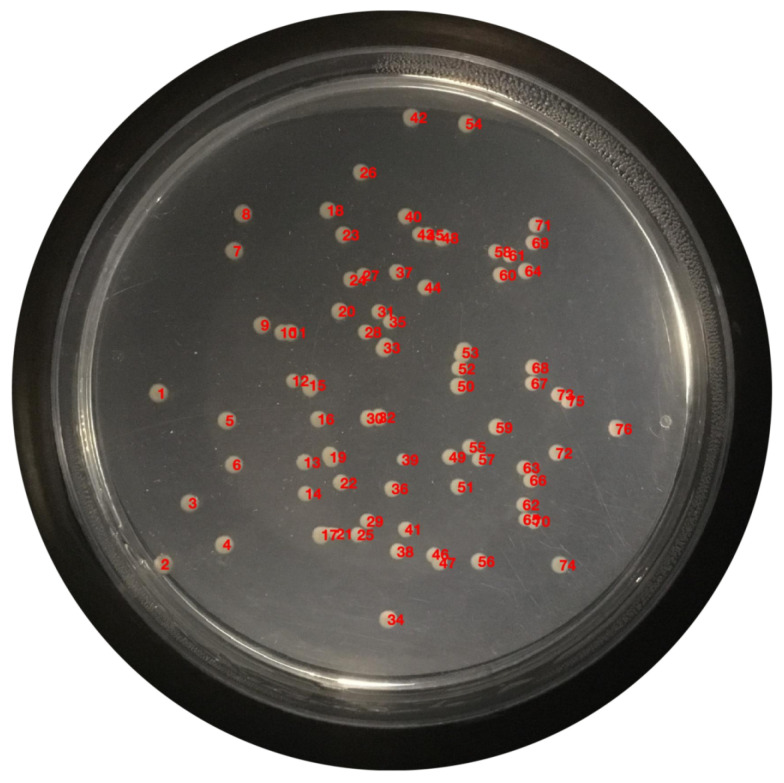
Enumerated colonies on the original image.

At this moment, the colonies are quantified; the enumeration is performed and displayed on the original image, exhibited in Figure 19.

**Figure 20:**
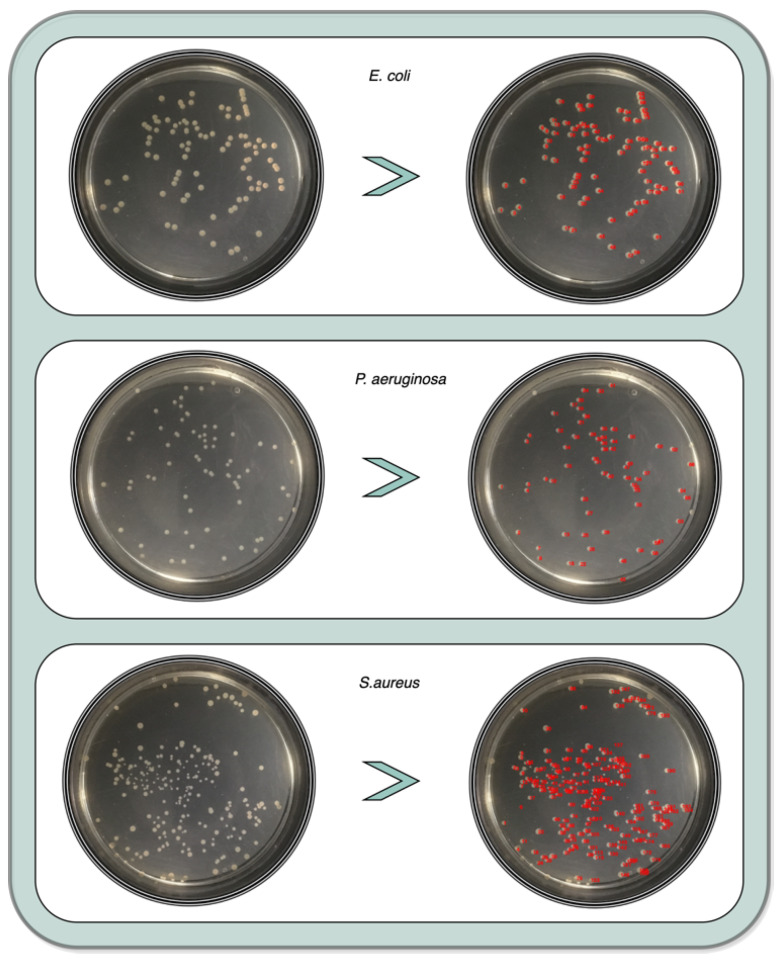
Enumerated example for the 3 colony study species.

Figure 20 shows an example of all the enumeration processes for the 3 colony study species.

### 2.6. Classification Measurements

After the enumeration stage, a comparison is made with the real label of each image; from the comparison, it is possible to compute the classification measures of the enumeration, i.e., *Accuracy*, *Recall* and *F-measure* [34] and consequently access the performance of the overall process.

## 3. Results and Discussion

The entire method has been described in detail in Section 2.3 where it is possible to understand step by step the applied semi-automatic enumeration process. All particular features were also specified in the abovementioned sections. To evaluate the efficiency of the developed process, the method has been tested in all the 1150 images collected for the study, and information has been gathered about each image in the collected database, specifically, manual counts, algorithm counts, false positives, false negatives and the statistical classification results—*accuracy*, *recall* and *F-measure*. From the aforementioned information, three tables were developed to enable a more detailed and comprehensive evaluation of the enumeration process.

Table 2 exhibits the *accuracy* results subdivided in 50-colony-spaced intervals, from [0–50] to “more than 300”. Concerning this last interval, it is worth mentioning why it was selected; the upper-limit, while referring to common acceptance for countable colonies on a plate, ranges from 250 to 300 [35], thus plates with more than 300 colonies were simply labeled as “more than 300”. Consequently, the *accuracy* on that group was computed by verifying if the given plate has in fact more than 300 colonies. If true, the attributed *accuracy* was 100%, i.e., if the algorithm performs an enumeration of 326 colonies on a “more than 300” plate (real label), it is considered 100% precise even if the plate itself contains more than 326 colonies (due to the mentioned reasons *recall* and consequently *F-measure* were not computed for this group).

Table 3 expresses similar information as the previous one, although the data is scattered in bigger classes, i.e., from [0–100] to [201–300].

In Table 4, *E. coli*, *P. aeruginosa* and *S. aureus* images were separately analyzed to evaluate the *accuracy* of the process concerning each species. To access the overall *accuracy*, the respective data was divided into two major classes: [0–300] and more than 300.

**Figure 21:**
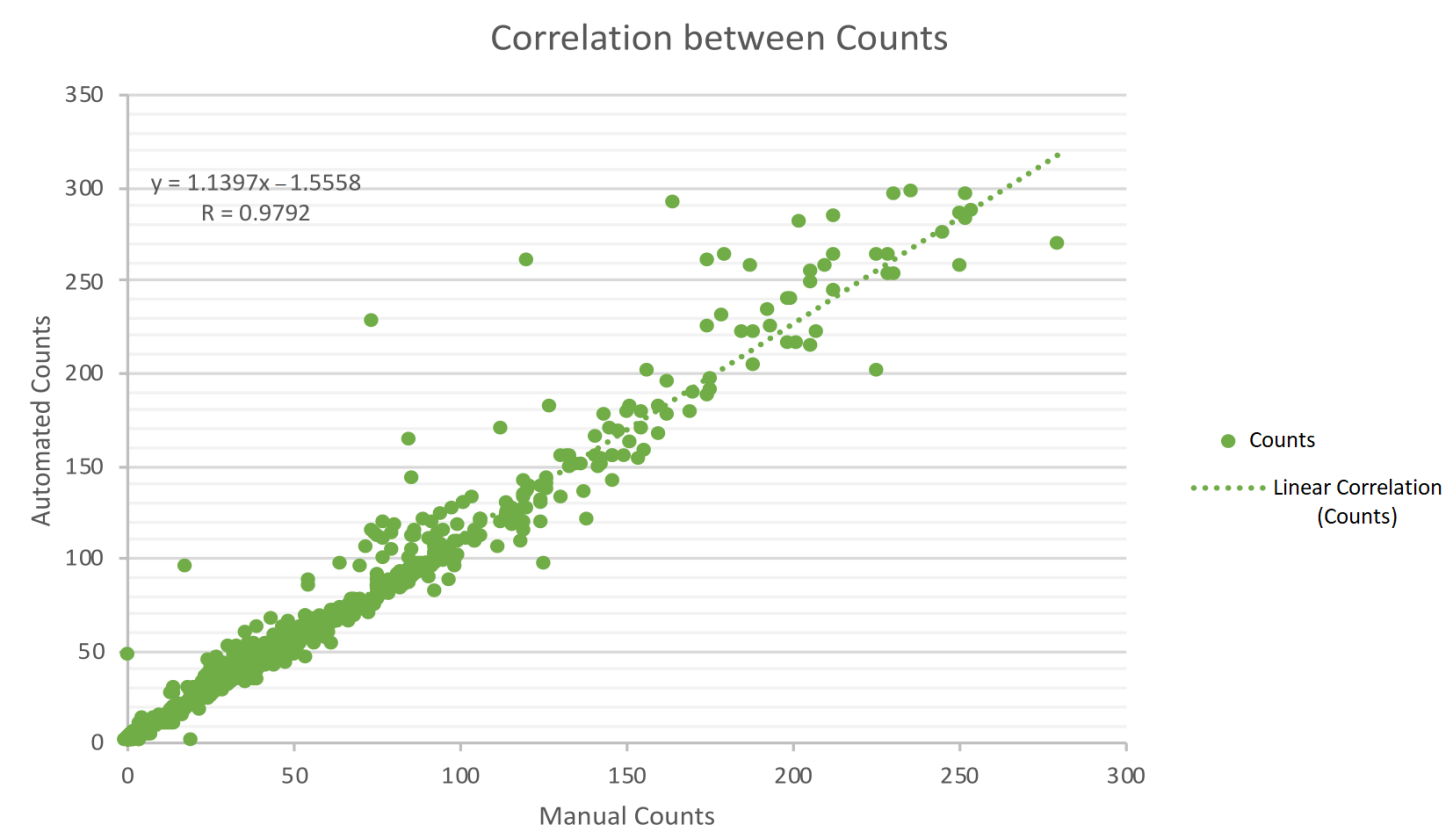
Graphical correlation between automated and manual counts.

From the analysis of Table 2 and Table 3, satisfying results are exhibited, roughly between 80% and 92%. Accuracy measures tend to decrease with increasing colony numbers, as expected. This latter circumstance is more perceptible in the “Others” class, a group formed by specifically catalogued and overcrowded plates. Nevertheless, in terms of relevance for a technician or a researcher, it is often enough to know that the plate is above 300 colonies [35]. Regarding the mentioned acknowledgement, the method demonstrates efficiency with 93% *accuracy* on the “more than 300” group. From Table 4, accuracy results of *E. coli* should be highlighted, evidencing values of 95%. In addition, Figure 21 shows the overall correlation (excluding “more than 300” class) between the automated and the manual counts, indicating a correlation coefficient (R) of 0.9792, thus meaning highly correlated data.

The parameters *accuracy* and *recall* obviously affect statistical results, where a large number of false positives and false negatives will lower *accuracy* and *recall*, respectively. In particular in this work, the false positives resulted from: condensation droplets on the plates’ walls, clumps derived from less effective spreading, agar defects in the Petri-plates, a small round-shaped mark inherent on each plate and residual noise resulting from incomplete elimination during the processing. The causes for the false negative cases are due to colonies located exactly on the rim of the plate which are eliminated during processing, and concatenated colonies are sometimes wrongly excluded because of their non-circularity or exaggerated area. Additionally, during the process the only input required of the user, i.e., selection of the minimum colony size, (at the beginning of the computation). If the colony is missed or wrongly selected, inconsistencies could occur in specific situations. Summarily, the enumeration system displays consistent accuracy which can be further reinforced by overcoming the abovementioned limitations.

As can be seen in Table 1, some studies report image analysis algorithms for bacterial enumeration by viable cell counting. The present work reports enumeration results for three colony types or bacterial species, while most works focus and “overfit” their algorithm just for one species which results in an algorithm with less flexibility for segmentation and enumeration for other species. The enumeration accuracy results achieved in the present work are at the same level as those reported in state-of-art works, but the conclusions should be carefully drawn as different databases were used to achieve the desired goal, and the image-based algorithm is also different.

Moreover, the speed of the developed algorithm hinges on the processing capacity of the computer used and on colony size. However, the below-presented estimation was obtained based on average values between minor and major computation time intervals. Speed tests were executed on an Intel^®^ i5 CPU running at 2.4 GHz. The execution times of the overall process ranges from 36 s to 44 s in 30 and 300 colonies, respectively. Table 5 reveals the approximate execution times of both manual and algorithm counts associated with the number of colonies in the plate; the selected number of colonies ranged from 30 to 300.

**Figure 22:**
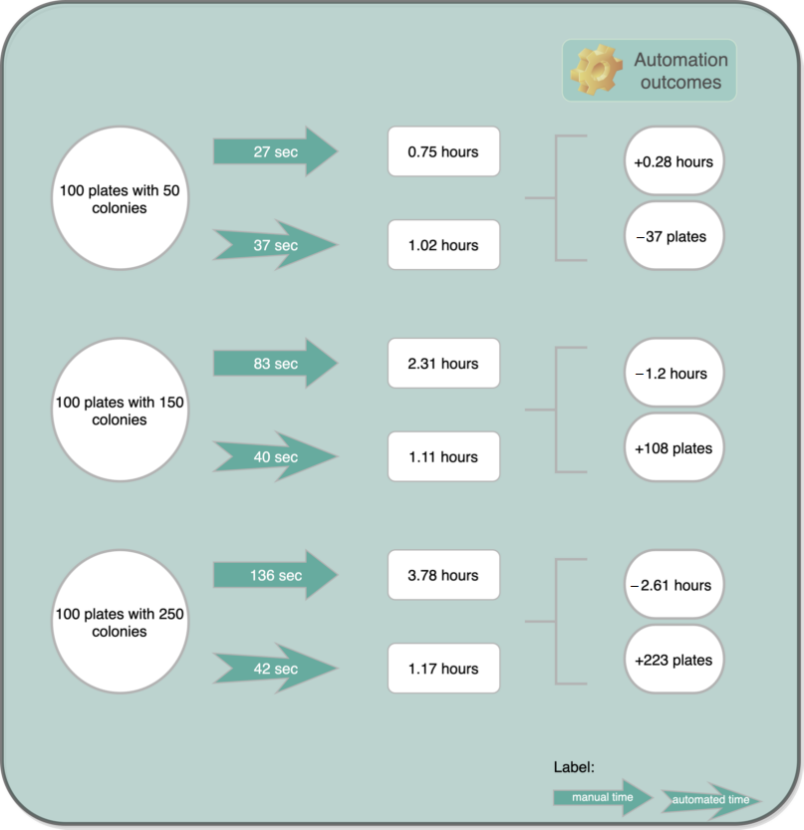
Exercise of time management with an automated enumeration process.

Based on Table 5, in terms of time management, an exercise could be carried out (represented in Figure 22) addressing some implications of an adopted automated enumeration procedure.

As can be seen in Figure 22, the proposed counting system is capable of reducing the manpower as well as lowering the time required for counting colonies in half and in one third for some situations, resulting in improved efficiency and productivity for the technician or the researcher.

## 4. Conclusions

There are numerous commercially available instruments that perform plate enumeration with various advantages: saving costs by reducing enumeration time, automatic documentation, reproducibility, and operator independence. However, despite the advantages of automated image analysis, it is still necessary to invest in an expensive, high performance commercial system, or to acquire expert knowledge in image processing. So, this work proposed and validated a semi-automated colony-counting low-cost system with low user-provided action. It was demonstrated to be capable of counting the number of *E. coli*, *P. aeruginosa* and *S. aureus* within acceptable rates of accuracy. Regarding values of *accuracy*, *recall* and *F-measure* for: (1) *E. coli*, the mean were 95%, 95% and 0.95, respectively; (2) *P. aeruginosa* were 91%, 91% and 0.90, respectively; and (3) *S. aureus* were 84%, 86% and 0.85, respectively. Thus, according to the results, *E. coli* is the most-efficiently-detected strain. The overall and mean values of *accuracy*, recall and *F-measure* were 91%, 92% and 0.92; 88%, 90% and 0.89, as well as 82%, 86% and 0.84, categorically split in [0,100], [101,200] and [201,300] intervals. The results of the more than 300 colonies category were very satisfactory with a mean *accuracy* value of 94%. In addition, *E. coli*, *P. aeruginosa* and *S. aureus* are round in shape; therefore, the proposed counting approach can be applied to any bacterial strain that develops round-shaped colonies. Dry cracks in the agar, poorly performed spreading techniques and illumination inconsistencies might lead to difficulties in the processing of the images and should therefore be avoided during culturing and image acquisition. However, since during image acquisition the Petri-plate was illuminated using back-lighting, images acquired from plates with non-transparent media might not be easily captured by the employed apparatus. Furthermore, the image processing procedures were developed based on features of colonies with transparent media; therefore media with diverse colors and opacity might yield unsatisfactory outcomes.

## Figures and Tables

**Table 1:** State-of-the-art image analysis-based methods.

Microorganism Type	Pre-Processing	Segmentation	Accuracy	Work
Bacteria	HSI color space processing	Thresholding	90%	[18]
Bacteria	-	Iterative thresholding and Hough Transform	86.76%	[19]
Bacteria	Median Filter	Thresholding, Canny Operator and Hough Transform	92.31%	[20]
Bacteria	Laplacian Filtering	Watershed and Distance Transform	90.30%	[21]
Bacteria	Laplacian Filtering and Hough transform	Otsu thresholding	90%	[22]
Bacteria	-	Watershed	80%	[23]
Bacteria	Median Filter	Distance Transform and Watershed	86.5%	[24]
Bacteria	Contrast limited adaptive histogram equalization	Watershed	92.1%	[25]
Bacteria	-	Thresholding	92.8%	[26]
Bacteria	Morphological filter	Random Hough circle transform and thresholding	92.1%	[27]

**Table 2:** *Accuracy*, *recall* and *F-measure* results discriminated into seven groups.

Interval	*Accuracy*	*Recall*	*F-Measure*
0–50	90%	91%	0.91
51–100	92%	93%	0.93
101–150	87%	89%	0.88
151–200	88%	90%	0.89
201–250	84%	87%	0.85
251–300	81%	86%	0.82
More than 300	94%	−	−
Others	74%	80%	0.77

**Table 3:** *Accuracy*, *Recall* and *F-measure* results discriminated into three groups.

Group	*Accuracy*	*Recall*	*F-Measure*
0–100	91%	92%	0.92
101–200	88%	90%	0.89
201–300	82%	86%	0.84
More than 300	94%	−	−

**Table 4:** *Accuracy*, *recall* and *F-measure* results sorted by bacterial species.

Bacteria	*Accuracy*		*Recall*	*F-measure*
	0–300	More than 300	0–300	0–300
*E. coli*	95%	96%	95%	0.95
*P. aeruginosa*	90%	93%	91%	0.90
*S. aureus*	84%	94%	86%	0.85

**Table 5:** Response time tests of the enumeration algorithm.

# Colonies	t_manual_ (s)	t_algorithm_ (s)
30	20	36
50	27	37
100	58	38
150	83	40
200	109	41
250	136	42
300	162	44

## Data Availability

The data presented in this study are openly available in FigShare at doi, reference number 10.6084/m9.figshare.20109377.
